# Commentary: Complete Cranial Iliac Osteotomy to Approach the Lumbosacral Foramen

**DOI:** 10.3389/fvets.2017.00106

**Published:** 2017-07-03

**Authors:** Francois-Guillaume Saulnier Troff, Luca Motta, Virginie De Busscher

**Affiliations:** ^1^Soft Tissue/Orthopaedics/Neurosurgery Department, Veterinary Emergency and Specialty Hospital, Singapore; ^2^Northwest Surgeons, Frodsham, United Kingdom; ^3^Diagnostic Imaging Department, Veterinary Emergency and Specialty Hospital, Singapore

**Keywords:** foraminotomy, transiliac, ilium, lumbosacral region, dogs

My co-authors, Dr. Motta, Dr. De Busscher, and myself would like to thank Dr. Dyall and Dr. Schmoekel for their interesting contribution and interest in a novel approach to the lumbosacral spine that we reported in six dogs in 2014 (1). The original technique of a lumbosacral lateral foraminotomy through an iliac osteotomy required an osteotomy based on the same landmarks as in this case report (2). The main difference was that our technique spared the ventral iliac spine by performing an incomplete osteotomy of the ilium in a dorsoventral direction of just enough length to allow ventrolateral reflection of the cranial aspect of the ilium (Figure 1). While allowing adequate exposure to achieve decompression of all three zones of the foramen, as well as a partial lateral corpectomy, the main advantages of this novel technique were to perform minimal elevation of the middle gluteal muscle and allow stabilization of the osteotomy using two screws and a simple cerclage wire placed dorsally, thereby reducing the cost of the implants and facilitating implant removal should a repeat MRI be required at a later stage. In our preliminary study, one dog developed neurological signs on the controlateral limb and removal of the screws and cerclage wire was achieved with a minimal approach prior to a repeat MRI. An additional advantage of our technique was that single session bilateral procedure could be performed with the patient placed in sternal recumbency.

**Figure 1:**
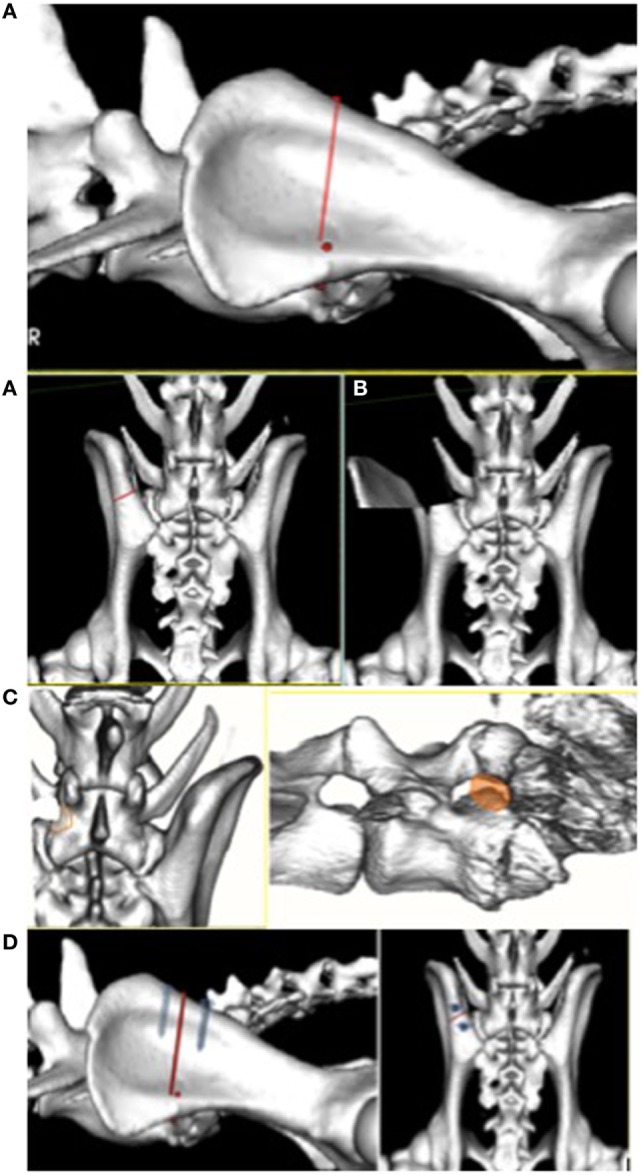
(From left to right, top to bottom) **(A)** Dorsoventral osteotomy of the ilium sparing the ventral iliac spine. **(B)** Ventrolateral reflection (70°–90°) of the cranial ilium exposing the stenotic foramen. **(C)** Tri-dimensional reconstruction 6 months after foraminotomy showing the neoforamen, cranioventral to the original foramen (orange shade). **(D)** Two titanium screws and a tension band are applied dorsally to stabilize the osteotomy after foraminotomy.

Our understanding when reading the discussion of this recent article is that the authors did not understand the principle of our technique nor accurately reported it in the discussion. Rather than “removing the dorsal part of the iliac wing” as stated by the authors in the discussion, our osteotomy was incomplete and stopped as soon as the mobility of the cranial ilium would allow adequate retraction laterally, typically leaving a third or a fourth of the dorsoventral length of the ilium intact. Conversely to what was reported in this case report, the cranial ilium could be freely retracted laterally while still preserving some ventral fibrous support in all clinical cases in our study. When adequately performed, the only tissues remaining that obscure the foramen would be the epaxial muscles, which still required retraction. In that respect, we are not sure that a more aggressive exposure and expensive stabilization procedure would be justified in most cases.

The technique reported in 2014 has since been applied on a larger population of dogs, with an overall follow-up period of over 2 years. Although these data are not yet published, the delay in reporting was necessary to gather enough objective follow-up data and assess the risk of recurrence through new bone formation. The technique was applied on 13 dogs, some had bilateral procedures, and our conclusion on a larger population remains unchanged—a lateral foraminotomy after ventrolateral reflection of the cranial aspect of the ilium provides an adequate approach to the lateral aspect of the L7-S1 intervertebral disc, the seventh lumbar nerve root, and all three zones of the lumbosacral foramen in a repeatable manner, irrespective of signalment. The original technique was designed with the assumption that we should not address a lumbosacral instability by causing more instability by performing a dorsal laminectomy and discectomy. Furthermore, we concluded that a lateral foraminotomy using an osteotomy of the ilium should not be combined with a dorsal laminectomy as it may predispose to fracture of the pedicle of the caudal articular facet of the seventh lumbar vertebra. This concern had previously been raised in our preliminary study and was confirmed with 2 out of 13 dogs (in our long-term follow-up) that had been treated years earlier with a dorsal laminectomy and underwent a foraminotomy at a later stage. Both dogs developed a non-displaced fracture of the pedicle that was documented on follow-up computed tomography imaging at 6 weeks and 6 months. If the procedure had been applied bilaterally, this fracture could have led to major complications. Fractures of L7 vertebral articular facets and pedicles were previously described by Moens and Runyon (3) following a standard dorsal laminectomy. Similar concerns were raised by Schwarz (4) when using a dorsolateral foraminotomy, previously described by Gödde and Steffen (5), in combination with dorsal laminectomy. The authors reported two dogs with postoperative fractures of the articular processes and subsequent painful instability. Both dogs were large-breed dogs. One was euthanized, the second one required surgical stabilization. A lateral foraminotomy combined with a dorsal laminectomy weakens the pedicle both medially and ventrally and could lead to fracture of the caudal articular facet of L7. The osteotomy described by Dyall and Schmoekel (2) is more extensive than the technique that we previously reported, more costly in implants, and despite the minor variations in their technique there is little to suggest this would improve the biomechanical effects of a lumbosacral foraminotomy combined with a dorsal laminectomy. Therefore, we are concerned that the readers could underestimate the risk of L7 caudal articular facet fractures on the basis of this single case report.

In conclusion, we would invite the readers to read the original description of the technique in six dogs (1) and formulate their own opinion. A long-term study with over 2 years follow-up over a larger population of dogs should soon provide more hindsight on treatment of lumbosacral foraminal stenosis using an incomplete iliac osteotomy.

## Author Contributions

F-GT wrote the commentary. VB and LM took part to the original technique reported and reviewed the commentary. Both agreed for submission of the General Commentary.

## Conflict of Interest Statement

The authors declare that the research was conducted in the absence of any commercial or financial relationships that could be construed as a potential conflict of interest.
